# Combination of Superheated Steam with Laccase Pretreatment Together with Size Reduction to Enhance Enzymatic Hydrolysis of Oil Palm Biomass

**DOI:** 10.3390/molecules23040811

**Published:** 2018-04-02

**Authors:** Nur Fatin Athirah Ahmad Rizal, Mohamad Faizal Ibrahim, Mohd Rafein Zakaria, Ezyana Kamal Bahrin, Suraini Abd-Aziz, Mohd Ali Hassan

**Affiliations:** 1 Department of Bioprocess Technology, Faculty of Biotechnology and Biomolecular Sciences, Universiti Putra Malaysia, 43400 UPM Serdang, Malaysia; nurfatinrizal@gmail.com (N.F.A.A.R.); mohdrafein@upm.edu.my (M.R.M.Z.); ezyana@upm.edu.my (E.K.B.); suraini@upm.edu.my (S.A.-A.); alihas@upm.edu.my (M.A.H.); 2 Laboratory of Biopolymer and Derivatives, Institute of Tropical Forestry and Forest Products, Universiti Putra Malaysia, 43400 UPM Serdang, Malaysia

**Keywords:** oil palm biomass, physical pretreatment, biological pretreatment, lignin removal, lignocellulosic biomass

## Abstract

The combination of superheated steam (SHS) with ligninolytic enzyme laccase pretreatment together with size reduction was conducted in order to enhance the enzymatic hydrolysis of oil palm biomass into glucose. The oil palm empty fruit bunch (OPEFB) and oil palm mesocarp fiber (OPMF) were pretreated with SHS and ground using a hammer mill to sizes of 2, 1, 0.5 and 0.25 mm before pretreatment using laccase to remove lignin. This study showed that reduction of size from raw to 0.25 mm plays important role in lignin degradation by laccase that removed 38.7% and 39.6% of the lignin from OPEFB and OPMF, respectively. The subsequent saccharification process of these pretreated OPEFB and OPMF generates glucose yields of 71.5% and 63.0%, which represent a 4.6 and 4.8-fold increase, respectively, as compared to untreated samples. This study showed that the combination of SHS with laccase pretreatment together with size reduction could enhance the glucose yield.

## 1. Introduction

Lignocellulosic biomass, produced mainly from agricultural industry and forestry wastes, is the most abundant plant material on Earth. There has been increasing interest in utilizing this lignocellulosic biomass in recent years due to its potential to be used as fermentation substrate for various valuable products, including biofuels and bio-based chemicals [1]. In Malaysia, oil palm biomass is the most abundant plant material generated every year, since palm oil is the biggest Malaysian agricultural commodity. In 2016, this crop occupied a total of 5.74 million hectares of Malaysia’s land with the production of 17.32 million tonnes of crude palm oil [2]. Processing of palm oil from fresh fruit bunch (FFB) at the mills generates 7.34 million tonnes of oil palm empty fruit bunch (OPEFB), 7.72 million tonnes of oil palm mesocarp fiber (OPMF), 4.46 million tonnes of oil palm kernel shell (OPKS) and 64 million tonnes of palm oil mill effluent (POME) per year [3]. The OPEFB and OPMF, which are the most abundant oil palm biomass forms generated at the mills, has not yet been fully utilized. It is currently either being used as mulching at plantations or dumped at the nearby factories for natural degradation. Recently, both materials have been commercialized for biocompost [4], biochar and activated carbon production [5]. These materials also have been tested for various fermentation processes including biobutanol [1], bioethanol [6], biohydrogen [7] and many more. However, the major concern while utilizing these biomasses as feedstock for fermentation is the effectiveness of the conversion into fermentable sugars.

OPEFB and OPMF are composed of 60–75% [8,9] and 50–55% [10,11] of cellulose and hemicellulose, respectively. These sugar polymers can be hydrolyzed into sugar monomers which subsequently can be used as substrates for fermentation. Like other lignocellulosic biomasses, OPEFB and OPMF are also composed of lignin that protects cellulose and hemicellulose and hinders enzymatic hydrolysis into sugars by cellulase. Generally, lignin is the most complex structure and represents about 10–25% of the biomass weight [8,9,10,11]. It is a long chain and heterogenous polymer, composed of mostly phenylpropane units, linked by ether bonds [12]. It has aromatic and rigid biopolymer properties linked via covalent bonds to xylans. The lignin structure inside the plant cell wall makes lignocellulosic biomass more rigid and highly compact.

In order to utilize oil palm biomass as a fermentation substrate, suitable and effective pretreatments are required to reduce the recalcitrance of lignocellulosic biomass by extensive modification of its lignocellulosic structure, especially of lignin [13]. The modification process can be carried out using physical, physico-chemical, chemical and/or biological pretreatments [12]. Although chemical pretreatment using either alkali or acid has been reported as the most effective pretreatment to generate high sugar yields in a short time [14], this approach may cause negative impacts on the environment, especially water pollution when it is released into the water stream. Therefore, in order to make sure that lignocellulosic biomass is reliable as a fermentation substrate, combinations of chemical-free pretreatments should be explored and proved as being effective, clean and feasible on an industrial scale.

Superheated steam (SHS) is a type of steam pretreatment that has been reported as a good pretreatment to breakdown and loosening the structural arrangement of lignocellulosic components in biomass [15,16,17]. SHS is a dry steam that is produced by adding heat to wet steam. The additional heat aids in raising the saturated steam temperature to exceed the boiling point of the liquid at a certain pressure [15]. The lignocellulosic material exposed to a high steam temperature of more than 180 °C can degrade the hemicellulose components since hemicellulose is less thermally stable than lignin and cellulose. Degradation of hemicellulose reduces the recalcitrance of the lignocellulosic material. It should also be noted that SHS is safe to be used since it can be conducted at atmospheric pressure with low energy consumption of 3.30 kW, and could cause very little environmental impact if collected condensate is reused [16]. However, pretreating the lignocellulosic biomass using SHS resulted in a low sugar yield after the saccharification process [17].

Combining biological pretreatment after SHS could improve the whole pretreatment process to produce sugars. Biological pretreatment of lignocellulosic biomass can be carried out by applying microorganisms (microbial pretreatment) or ligninolytic enzymes (enzymatic pretreatment) to digest lignin components. Enzymatic pretreatment is faster than microbial pretreatment, hence the process is also easier to control. In addition, it requires only mild conditions and the process specifically only attacks the lignin [18,19]. Laccase (EC 1.10.3.2; benzenediol:oxygen oxidoreductase) is an oxidizing enzyme that was extensively studied for lignocellulosic biomass pretreatment [14,16]. It is a multicopper oxidase produced by fungi, plants and bacteria to specifically degrade lignin components. The oxidation of a laccase substrate leads to the formation of free radicals and reduction of molecular oxygen into water molecules [18,20]. However, laccase pretreatment alone does not produce a high yield of hydrolyzed sugars [21,22]. Therefore, combining this enzymatic pretreatment using laccase with SHS could enhance the saccharification performance of oil palm biomass into sugars. In addition, the effect of size reduction prior to laccase pretreatment was also conducted since the enzyme action is highly affected by the exposed surface area of the substrate.

## 2. Results and Discussion

### 2.1. Chemical Composition Analysis

The chemical compositions of raw OPEFB and OPMF in a dry basis are shown in Table 1. All the chemical components of OPEFB analyzed in this study are comparable with the results previously reported by Zakaria et al. [8]. However, the hemicellulose component was slightly lower as compared to the report of Kong et al. [9]. The value of cellulose, hemicellulose and acid insoluble lignin of OPMF are comparable with Zakaria et al. [10], but lower as compared with Iberahim et al. [11]. The variations of the chemical compositions might be due to the different factors affecting the collected samples, such as plantation area, planting batch, maturity level and year [23]. Besides, the compositional methods that have been employed might also contribute to the variation of chemical composition obtained [10]. Therefore, it is very important to tabulate the chemical compositional analysis for every experiment that was conducted since the total carbohydrates and lignin contents make up a major portion in biomass, and these constituents must be determined as a part of a comprehensive biomass analysis. Comparing between OPEFB and OPMF, results of this study showed that the total carbohydrates in OPEFB were 59.4%, which is higher than OPMF (51.8%). Lignin composition of OPMF was 31.3%, which is higher than OPEFB (25.6%), contributing to a tougher structural arrangement than OPEFB.

### 2.2. Effect of Pretreatments on Chemical Compositions

#### 2.2.1. Superheated Steam Pretreatment

The superheated steam (SHS) pretreatment acts as an initial pretreatment to open up the structure of biomass. The recalcitrance of the lignocellulosic material becomes loosened as the hemicellulose is solubilized when OPEFB and OPMF are exposed to a temperature of 180 °C for 60 min and 190 °C for 60 min, respectively. Degradation of hemicellulose can be observed in both samples of OPEFB and OPMF, with an 18.7% and 21.3% reduction of hemicellulose percentage, respectively (Table 2). Hemicellulose has a side chain (branched) and backbone that are sensitive to thermal processes [24]. High temperature causes the degradation of acetyl groups in hemicellulose in the form of acetic acid, while pentose and hexose sugars degrade into furfural and 5-HMF. Therefore, hemicellulose has a higher degree of depolymerization than cellulose and lignin. High reduction of hemicellulose after pretreatment with SHS caused an increment of the lignin and cellulose compositional percentage, but the lignin that interact while the hemicellulose was loosened up and this makes the structural arrangement weaker. This observation was supported by the increased of glucose yield after the saccharification of the SHS-pretreated sample. A slight increment of glucose yield equivalent to 18.4% for OPEFB and 15.6% for OPMF might be a result of the formation of pseudo-lignin from carbohydrate (hemicellulose) degradation that migrated to the surface of biomass and is deposited as lignin droplets [25,26]. This formation also resulted in an increment of total lignin composition in both SHS-pretreated OPEFB and OPMF.

#### 2.2.2. Effect of Laccase Loadings on Lignin Removal

Several experiments were conducted to determine the most suitable laccase loading for lignin degradation in OPEFB and OPMF. Both samples were treated with laccase loadings ranging between 20–100 U/g-substrate. However, the results show that OPMF pretreated with these laccase loadings does not lose any lignin. This situation might be due to a higher lignin composition in OPMF, and because its structural arrangement is tougher and more rigid than that of OPEFB. Therefore, a higher range of laccase loading (100–800 U/g-substrate) was applied to delignify OPMF and the results are tabulated in Table 3. These results showed that laccase loading had a significant effect on lignin removal for both OPEFB and OPMF. The lignin removal for OPEFB was improved from 3.5% to 10.9% when the laccase loading increased from 20 to 100 U/g-substrate. There is no further lignin removal observed when a laccase loading of more than 100 U/g-substrate was added. Meanwhile, OPMF has maximum lignin removal of 8.3% at a laccase loading of 400 U/g-substrate. It can be observed that there was a gradual decrease in the lignin removal percentage with the increase of laccase concentration until it reached the enzyme saturation point. Delignification by laccase occurs when the substrates are oxidized with the reduction of oxygen to water, which generates free radical electrons [18]. This experiment showed that the lignin composition and structural arrangement of lignocellulosic biomass could affect the amount of laccase needed for the pretreatment.

In comparison with other studies, this experiment showed an improved delignification of lignocellulosic biomass as shown in Table 4. The previous study by Zanirun et al. [22] reported that OPEFB pretreatment with 50 U/g-substrate of laccase by *Pycnoporus sanguineus* UPM4 removed 3.1% of the lignin, which is lower than the lignin removal presented in this study. A sufficient amount of laccase loading is important to improve the removal of lignin components in lignocellulosic biomass. Compared with other types of biomass, wheat straw fiber pretreatment using 65 U/g-substrate of laccase loading from *Pycnoporus cinnabarinus* removed only 5.0% of the lignin [27]. A small reduction of lignin content (1.3%) has also been observed when furfural residues were pretreated using 100 U/g-substrate of laccase loading from *T. versicolor* [28]. Pretreatment of an *Eucalyptus globules* kraft pulp with 17.5 U/g-substrate of laccase from *T. vilosa* resulted in 23% lignin removal [29].

In addition, a higher percentage of lignin removal was observed when the OPEFB and OPMF samples were ground to a size of 0.25 mm. It should be noted that there are limited reports on delignification of OPMF through biological pretreatment by either microbial or enzymatic pretreatment. Beside the lignin removal, the polysaccharide compositions were also evaluated as shown in Table 2.

The cellulose composition of OPEFB and OPMF increased to 45.5% and 36.8%, respectively, as compared to the untreated biomass. On the other hand, the percentage of hemicellulose was reduced by 3.8% for OPEFB, and 5.4% for OPMF. The recalcitrance of the biomass was further reduced with the greater losses of lignin.

#### 2.2.3. Total Phenolic Compounds after Laccase Pretreatment

**Figure 1:**
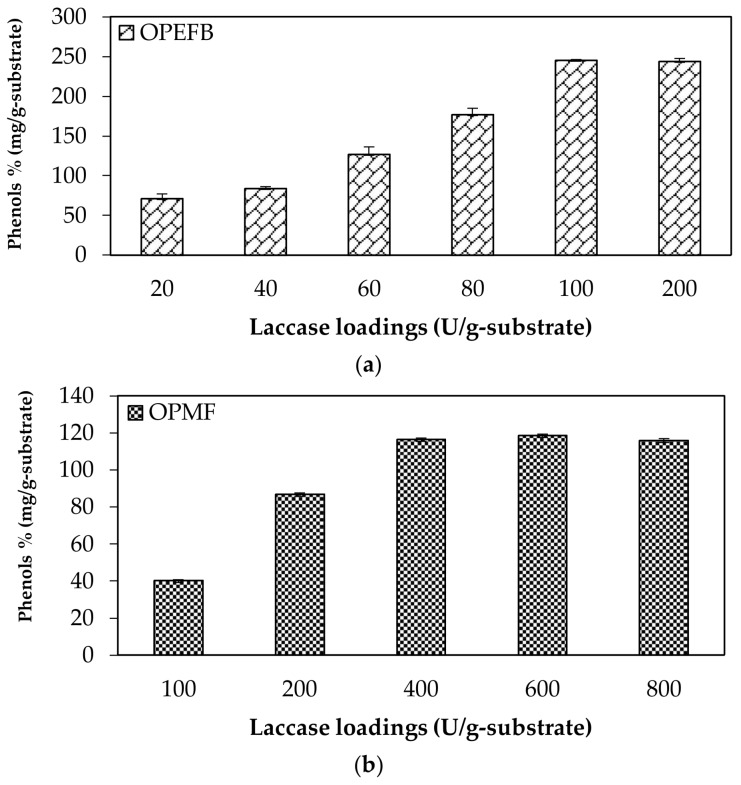
Total phenolic compounds after laccase pretreatment on (**a**) oil palm empty fruit bunch (OPEFB) and (**b**) oil palm mesocarp fiber (OPMF).

To evaluate the effect of different laccase loadings on the removal of phenolic compounds from lignin components, the concentration of total phenolic compounds was measured and the degradation products were recovered in the liquid fraction after the pretreatment. Phenols are released due to partial solubilization and degradation of the lignin during the pretreatment [18,19]. The concentration of total phenolic content obtained after laccase pretreatment of OPEFB and OPMF with different laccase loading is shown in Figure 1.

Phenols have inhibitory effects on saccharification and fermentation processes by inhibiting the activities of cellulolytic enzymes and microbes, thus, decreasing yields and lowering the productivity. Phenols also can alter the growth of fermenting microorganisms [19]. Evaluation of the laccase loading for OPEFB showed that the concentration of total phenolic content increased gradually until the concentration become constant at a laccase loading of more than 100 U/g-substrate, with a phenol concentration of 245.17 mg/g. Meanwhile, in OPMF, the highest phenolic compound content was 116.46 mg/g after pretreatment using 400 U/g-substrate and become constant at higher laccase loadings. These results were compatible with the total lignin removal obtained as presented in Table 3, where the lignin degradation reflects the total quantified phenolic compounds. Total phenolic compound is an indicator to verify the concentration of phenolic compounds present, in which it is also related to the structure, reactivity and mechanism of lignin degradation [18,20]. Laccase catalyzes the oxidation of phenols and form unstable phenoxy radicals. These radicals can interact with each other and contribute to destroying aromatic compounds [20].

#### 2.2.4. Combination of Pretreatments with Size Reduction

OPEFB and OPMF pretreatment using SHS + laccase has been conducted to examine the suitability of this combination. Results showed that OPEFB (raw size) pretreated using SHS + laccase had an increased cellulose percentage from 38.1% (untreated) to 47.4%, with lignin removal of 17.6%. It can be clearly observed that the cellulose composition was increased when the OPEFB was pretreated using SHS only, followed by laccase only, and SHS + laccase as shown in Table 2. Similar situations can be observed for OPMF that followed the same trend as OPEFB. However, a lower lignin removal percentage was observed might be due to a tougher structural arrangement than in case of OPEFB.

In order to improve the enzymatic pretreatment by laccase, the SHS pretreated OPEFB and OPMF were ground to 2, 1, 0.5 and 0.25 mm using a hammer mill and delignified by laccase at 100 U/g-substrate for OPEFB, and 400 U/g-substrate for OPMF. Reduction of size from raw to 0.25 mm had significantly increased the lignin removal of OPEFB up to 38.7% and increased the cellulose composition to 57.3%. A similar trend was observed for OPMF, where the cellulose composition increased from 39.3% (raw size) to 49.3% (0.25 mm) with 39.6% of lignin removal. Both OPEFB and OPMF showed greater lignin removal when the substrate size was reduced from raw to 0.25 mm. Small particle size increases the total surface area, homogeneity and heat transfer efficiency [30,31,32]. Therefore, enzymatic digestibility by laccase has been improved by increasing the surface area of the substrate.

Although the enzyme action could be enhanced by reducing the substrates’ size to less than 0.25 mm, the milling process using a hammer mill consumes more energy to generate smaller particle sizes. According to Ndukwu et al. [32], the specific energy requirement (kWht^−1^) to grind palm kernel using a hammer mill to a size of 5–0.8 mm consumes 0.2–2.3 kWh of energy. However, the hammer mill has been reported as a convenient and probably the most commonly used method in order to obtain a suitable substrate size for subsequent processing [31,32]. In addition, it should be noted that particle size of less than 0.25 mm is not suitable for the pretreatment process because it may result in a low bias for carbohydrate and high bias for lignin content due to excessive carbohydrate degradation [33].

### 2.3. Structural Analysis Using SEM

**Figure 2:**
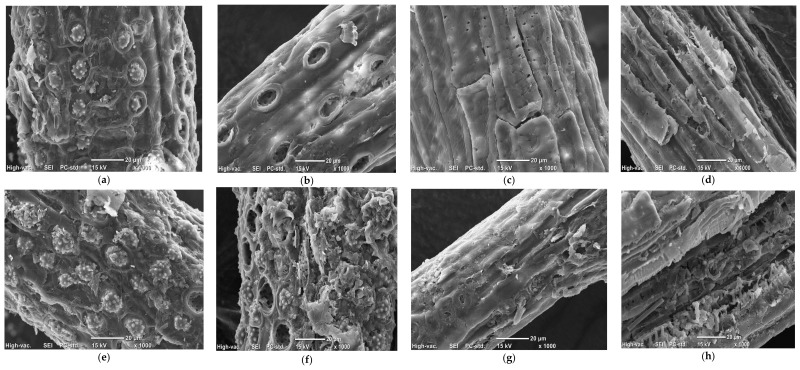
Scanning electron micrographs of oil palm empty fruit bunch (OPEFB) (**a**) untreated (**b**) SHS (180 °C, 60 min) (**c**) laccase (100 U/g-substrate) (**d**) SHS + laccase at 0.25 mm size and oil palm mesocarp fiber (OPMF) (**e**) untreated (**f**) SHS (190 °C, 60 min) (**g**) laccase (400 U/g-substrate) and (**h**) SHS + laccase at 0.25 mm size.

Scanning electron microscope (SEM) images were taken to investigate the morphological changes of OPEFB and OPMF after pretreatment, as shown in Figure 2. SEM images showed similar fiber-like structures containing silica bodies for both OPEFB and OPMF. The untreated OPEFB and OPMF had a rough surface on the whole area of the fiber (Figure 2a,e). Therefore, the structure of untreated OPEFB and OPMF displayed a rigid and highly ordered fibrils arrangement. A great amount of silica bodies that attached to circular craters over the strand surface of the fibers could also be observed in both the OPEFB and OPMF SEM images.

Based on the SEM images, the SHS pretreatment was able to remove the silica bodies from the structure and empty craters can be clearly observed in both substrates (Figure 2b,f). This observation indicates that sufficient energy from SHS was able to remove the silica bodies, hence the steam appeared can interrupt lignocellulosic materials beside its ability to loosen up the recalcitrance of the structure. The SEM images also showed that the surface of laccase-pretreated OPEFB and OPMF appeared to be more uniform and smooth, and all the silica bodies were removed. There were some cracks and formation of micropores that can be observed on the strands of the OPEFB and OPMF structure (Figure 2c,g). The microscopic alterations in the fiber have been generally considered as a result of lignin removal [34]. In Figure 2d,h, SHS and laccase-pretreated OPEFB and OPMF with particle size of 0.25 mm showed that the structural arrangements of the substrates have been altered and the outer layer of the fiber was ‘peeled off’. The degree of defibrillation and particle size reduction played a very important role to enhance the conversion of lignocellulosic material into hydrolyzed sugars.

### 2.4. Saccharification of Pretreated OPEFB and OPMF

**Figure 3:**
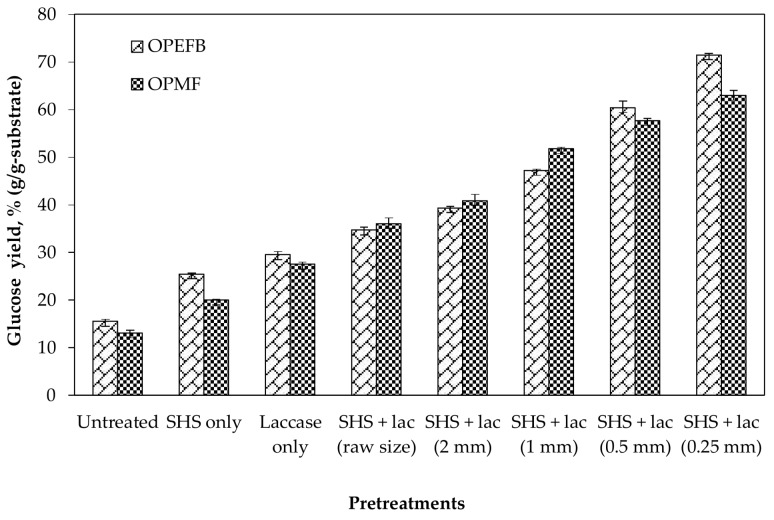
Hydrolysis performance of untreated and pretreated oil palm empty fruit bunch (OPEFB) and oil palm mesocarp fiber (OPMF). ‘SHS’—superheated steam, ‘lac’—laccase.

An efficient saccharification process is highly dependent on an effective pretreatment being applied to lignocellulosic biomass [12]. To investigate the efficiency of various pretreatments, the pretreated OPEFB and OPMF were subsequently submitted to a saccharification process using cellulase (Celluclast 1.5 L). This experiment showed that the glucose yield obtained for untreated OPEFB was only 15.5%, and 13.1% for untreated OPMF. The glucose yield was increased to 18.4% for OPEFB, and 15.6% for OPMF when treated with SHS alone. Meanwhile, substrates pretreated by laccase only generated up to 29.5% of glucose yield for OPEFB, and 27.5% of glucose yield for OPMF, which was higher than the SHS pretreatment. Higher degradation of lignin after laccase pretreatment than SHS pretreatment contributed to a higher digestibility of the cellulose structure into glucose. Several studies reported that lignin removal enhanced enzyme digestibility in the saccharification of lignocellulosic materials [8,18,27]. Besides, there was a little increment of lignin percentage observed after SHS pretreatment, which was due to attribution of pseudo-lignin that still adhered to the surface of the substrates, which constrains the saccharification process [25,26]. In this study, the glucose yield was further improved by reducing the substrate particle size from raw to 0.25 mm. The saccharification of OPEFB and OPMF pretreated with SHS + laccase at 0.25 mm size reduction as shown in Figure 3 resulted in the highest glucose yield of 71.5% and 63% for OPEFB and OPMF, respectively.

These values are equivalent to a 4.6-fold increment of the glucose yield for OPEFB, and a 4.8-fold increment for OPMF as compared with the untreated substrates. The efficiency of enzymatic hydrolysis was greatly improved due to the structural modification and lignin degradation of OPEFB and OPMF by combining SHS + laccase pretreatments, which made the cellulose more accessible to the cellulase. On top of that, reduction in particle size increased the surface area, and provided more accessible lignin components to be further degraded by laccase, and exposed more cellulose for enzymatic hydrolysis by cellulase. Based on these results, a combination of SHS + laccase pretreatment with size reduction to 0.25 mm enhanced the saccharification and increased the glucose recovery yield of both OPEFB and OPMF.

After 48 h of saccharification, the liquid fraction from OPEFB and OPMF were taken to quantify the inhibitory compounds using HPLC. The presence of inhibitory compounds in sugars could negatively affect the subsequent fermentation process. The main inhibitory components include furan derivatives, aliphatic acids, phenolic and other aromatic compounds [18,20]. Furfural and 5-HMF are generated from the furan derivatives derived from cellulose and hemicellulose and can be further degraded to form levulinic acid and formic acid. Hydrolysis of the acetyl groups in the hemicellulose generates acetic acid. Meanwhile, phenolic compounds like 4-hydroxybenzoic acid, 4-hydroxybenzaldehyde, vanillic acid, syringic acid, vanillin, syringaldehyde, *p*-coumaric acid, ferulic acid and coniferyl aldehyde are derived from the degradation of lignin [18,20]. However, these inhibitory compounds were not detected in the sugar produced in this study. This is because after the combination pretreatment of SHS + laccase, the whole slurry was filtered and washed. The purpose of washing the substrates after the pretreatment is to prevent the inhibitory compounds from affecting the saccharification.

## 3. Materials and Methods

### 3.1. Raw Materials

Pressed and shredded OPEFB and OPMF were obtained from Seri Ulu Langat Palm Oil Mill, Dengkil (Selangor, Malaysia). The OPMF was manually separated from the crushed kernels and shells to prevent errors in the experiments. The pressed and shredded OPEFB (10–50 mm) and OPMF (10–30 mm) were sun dried and stored in sealed plastic bags at room temperature prior to further use.

### 3.2. Characterization

#### 3.2.1. Determination of Extractives

The determination of extractives in OPEFB and OPMF were carried out according to the NREL laboratory analytical procedure [35]. The analysis was carried out using a two-step Soxhlet extraction. The cellulose thimble was weighed, and the samples were added to a cellulose thimble. The cellulose thimble was inserted into the Soxhlet tube and the round bottom flask containing 200 mL of deionized water. First, hot water extraction was carried out for 8 h to remove water-soluble compounds and nitrogenous material. After this process completed, the thimble was carefully removed and dried in an oven at 60 °C for 24 h and the thimble weight was measured. Second, the process was continued using another Soxhlet extraction with 200 mL of 95% ethanol for 8 h and the thimble was carefully removed and dried in an oven at 60 °C for 24 h. Triplicate samples were used, and average values were calculated.

#### 3.2.2. Determination of Lignocellulosic Compositions

The composition of cellulose, hemicellulose, acid insoluble lignin and acid soluble lignin in OPEFB and OPMF were determined according to NREL laboratory analytical procedures as described by Sluiter et al. [36]. Approximately 0.3 g of dried OPEFB and OPMF was placed into a glass vial and hydrolyzed in 72% (*w/w*) of H_2_SO_4_ at 30 °C for 60 min, and the slurry was further hydrolyzed in diluted 4% (*w/w*) of H_2_SO_4_ followed by autoclaved at 121 °C for 60 min. The samples were vacuum filtered, and the liquid sugars were filtered again using 0.22 µm of nylon membrane filter and analyzed using an HPLC instrument equipped with a refractive index detector (Shimadzu, Kyoto, Japan). The residue left on the filter paper was dried overnight in an oven at 105 °C. Final weight of the residual after acid hydrolysis was measured as acid insoluble lignin while its filtrate was measured as acid soluble lignin. Acid soluble lignin was determined using a UV-Vis spectrophotometer (Shimadzu) at the wavelength of 205 nm. Triplicate samples were used, and average values were calculated.

#### 3.2.3. Determination of Ash Content

The ash content in OPEFB and OPMF was determined based on the NREL laboratory analytical procedures reported by Sluiter et al. [37]. Triplicate samples of OPEFB and OPMF in porcelain crucibles were placed in a muffle furnace and heated at 575 °C for 4 h. After the heating process, the crucibles were removed from the furnace and cooled down to room temperature in a desiccator before weighing the crucibles and ash. The procedures were repeated until a constant weight of samples were obtained.

### 3.3. Superheated Steam Pretreatment

The OPEFB and OPMF were pretreated using a lab scale SHS oven (DC Quto, QF-5200C, Naomoto Corporation, Osaka, Japan) with treatment chamber dimension of 300 mm × 265 mm × 100 mm. The SHS oven consists of a stainless-steel heating chamber and a boiler. The heater power of the SHS oven and the steam flow rate were conducted at 6.6 kW and 4.95 kg/h, respectively. The selection of pretreatment condition using SHS was based on the best pretreatment condition reported. For OPEFB, the pretreatment was conducted at 180 °C for 60 min [15], and 190 °C for 60 min for OPMF [17]. The SHS pretreated samples were ground using a hammer mill (Hsiangtai CW-1, Taipei, Taiwan) to 2, 1, 0.5 and 0.25 mm for subsequent use.

### 3.4. Laccase Pretreatment

The OPEFB and OPMF were pretreated using enzyme laccase produced by *T. versicolor* (Sigma-Aldrich, St. Louis, MO, USA) with enzyme loading of 20–200 U/g-substrate for OPEFB and 100–800 U/g-substrate for OPMF. The pretreatment was carried out using 2.5% of substrate concentration mixed with 0.05 M of sodium acetate buffer (pH 4.8) and incubated in a rotary incubator operated at 150 rpm, 50 °C for 24 h [28]. All experiments were performed in triplicates. After incubation, the pretreated sample mixtures were filtered using filter papers (No. 1, Whatman, Maidstone, UK) and then washed with deionized water until a neutral pH was obtained. Then, the pretreated samples were oven dried at 60 °C for 24 h.

### 3.5. Saccharification

The saccharification was conducted using commercial cellulase (Celluclast 1.5 L) purchased from Novozymes (Bassvaerd, Denmark). The experiment was performed by adding 5% of substrate concentration in 0.05 M of sodium acetate buffer (pH 4.8) with 30 FPU/g-substrate of cellulase activity. The mixtures were incubated at 50 °C in a rotary incubator shaker at 200 rpm for 48 h [15]. Samples were taken from the mixture and centrifuged for 10 min at 10,000 rpm for sugar determination. All experiments were performed in triplicates and results were presented as an average value.

### 3.6. Analytical Procedures

Monomeric sugars from saccharification were analyzed using a HPLC equipped with a refractive index detector (RID-10A, Shimadzu) and a Rezex RCM-monosaccharide column (Phenomenex, Torrance, CA, USA) equipped with a Carbo-Pb micro-guard cartridge. The column oven was set at 80 °C and samples were eluted at 0.60 mL/min using deionized water as a mobile phase [38]. The enzymatic digestibility was represented by the sugar yield (%) calculated as of the formula below:

(1) $$Sugar\;yield\;\left(\%\right)=\frac{Weight\;of\;monomeric\;sugars\;after\;enzymatic\;hydrolysis\;\left(mg\right)\;}{\begin{array}{c} Weight\;of\;potential\;total\;monomeric\;sugars\;after\;sulfuric\;acid\;hydrolysis\\ of\;oil\;palm\;biomass\;\left(mg\right)\; \end{array}}\times 100$$

Laccase activity was measured using 2,2’azinobis-(3-ethylbenzenthiazoline-6-sulfonic acid) (ABTS) as a substrate by a UV-Vis spectrophotometer (Shimadzu) at 420 nm with molar extinction coefficient, ε = 36,000 M^−1^·cm^−1^ based on Bourbonnais et al. [39]. The kinetic of the graph slope values were calculated to determine the activities of enzyme in Unit per millilitre (U/mL). Total phenolic content of the liquid fraction was quantified according to the Folin–Ciocalteau method described by Makkar et al. [40]. The sample (0.5 mL) was put into a test tube with 0.25 mL of Folin-Ciocalteu reagent and 1.25 mL of sodium carbonate solution. All the samples were vortexed and the absorbance was measured after 40 min at wavelength of 725 nm. Furfural, 5-hydroxymethylfurfural (5-HMF), acetic acid and formic acid were analyzed using a HPLC equipped with a refractive index detector (RID-10A, Shimadzu) and a BioRad Aminex HPX-87H column (Bio-Rad, Hercules, CA, USA) with a Carbo-H micro-guard cartridge. The column oven was set at 65 °C and samples were eluted at 0.60 mL/min using 0.005 M of H_2_SO_4_ as a mobile phase [41]. The surfaces morphological images of raw and pretreated fibers were examined using a scanning electron microscopy (SEM) with JCM-6000 PLUS Neo Scope Bench top SEM (Jeol, Tokyo, Japan). The fiber was coated with Pt for 30 s using an ion sputtering system (Hitachi, Tokyo, Japan) prior to images observation. The instrument was operated at a beam voltage of 15 kV and with 1000× magnification.

## 4. Conclusions

The combination of chemical-free pretreatments using SHS followed by laccase was successfully performed. Reduction of substrate size from raw to 0.25 mm improved lignin removal of OPEFB and OPMF by 38.7% and 39.6%, respectively. This pretreatment increased the glucose yield by 71.5% and 63.0%, respectively, as compared to the untreated substrates. This present study revealed the suitability of combining SHS with laccase pretreatment together with the positive effect of particle size reduction of OPEFB and OPMF.

## Figures and Tables

**Table 1:** Compositional analysis of oil palm empty fruit bunch (OPEFB) and oil palm mesocarp fiber (OPMF) in comparison with previous studies.

Samples	Components (%)							References
	Cellulose	Hemicellulose	Acid Insoluble Lignin	Acid Soluble Lignin	Water Extractives	Solvent Extractives	Ash	
OPEFB	38.1 ± 0.9	21.3 ± 1.1	22.6 ± 0.1	3.0 ± 0.0	8.3 ± 1.0	1.1 ± 0.1 ^a^	3.5 ± 0.5	This study
	40.4 ± 2.4	20.2 ± 2.3	23.1 ± 0.5	-	-	2.5 ± 1.9 ^b^	5.9 ± 0.3	[8]
	38.3 ± 0.1	35.3 ± 0.1	22.1 ± 1.6	-	-	2.7 ± 1.3 ^a^	1.0 ± 0.1	[9]
OPMF	27.8 ± 0.5	24.0 ± 0.4	27.9 ± 0.1	3.4 ± 0.2	4.9 ± 0.3	7.4 ± 0.6 ^a^	3.3 ± 0.3	This study
	25.5 ± 1.7	25.7 ± 3.3	25.5 ± 0.5	-	-	11.4 ± 0.4 ^b^	5.8 ± 0.2	[10]
	28.8 ± 0.5	25.3 ± 0.7	28.9 ± 2.1	-	-	6.3 ± 0.5 ^a^	2.6 ± 0.3	[11]

‘-‘ not determined; ^a^ ethanol extractives; ^b^ acetone extractives.

**Table 2:** Chemical composition of untreated and pretreated oil palm empty fruit bunch (OPEFB) and oil palm mesocarp fiber (OPMF).

Samples	Chemical Components (%)					
	Cellulose	Hemicellulose	Acid Insoluble Lignin	Acid Soluble Lignin	Total Lignin	Lignin Removal
Untreated OPEFB	38.1 ± 0.9	21.3 ± 1.1	22.6 ± 0.1	3.0 ± 0.0	25.6	-
Raw size, SHS 180 °C 60 min	43.2 ± 0.9	17.3 ± 0.4	28.0 ± 0.1	4.0 ± 0.3	32.0	-
Raw size, laccase 100 U/g	45.5 ± 0.2	20.5 ± 0.3	19.6 ± 0.5	3.2 ± 0.0	22.8	10.9
Raw size, SHS 180 °C 60 min, 100 U/g laccase	47.4 ± 1.3	16.0 ± 0.4	17.8 ± 0.8	3.3 ± 0.2	21.1	17.6
2 mm, SHS 180 °C 60 min, 100 U/g laccase	49.7 ± 0.9	16.2 ± 0.7	15.4 ± 0.6	3.2 ± 0.0	18.8	27.4
1 mm, SHS 180 °C 60 min, 100 U/g laccase	52.5 ± 0.8	15.7 ± 0.9	14.5 ± 0.8	3.2 ± 0.3	17.7	30.9
0.5 mm, SHS 180 °C 60 min, 100 U/g laccase	54.0 ± 0.4	14.9 ± 1.3	14.0 ± 0.7	3.0 ± 0.3	17.0	33.6
0.25 mm, SHS 180 °C 60 min, 100 U/g laccase	57.3 ± 1.3	14.2 ± 1.8	12.7 ± 0.6	3.0 ± 0.0	15.7	38.7
Untreated OPMF	27.8 ± 0.5	24.0 ± 0.4	27.9 ± 0.1	3.4 ± 0.2	31.3	-
Raw size, SHS 190 °C 60 min	33.2 ± 0.6	18.9 ± 1.8	35.9 ± 0.6	3.8 ± 0.0	39.7	-
Raw size, laccase, 400 U/g	36.8 ± 0.4	22.7 ± 0.5	25.6 ± 0.1	3.1 ± 0.1	28.7	8.3
Raw size, SHS 190 °C 60 min, 400 U/g laccase	39.3 ± 0.3	18.6 ± 0.1	23.1 ± 0.5	3.4 ± 0.0	26.5	15.3
2 mm, SHS 190 °C 60 min, 400 U/g laccase	43.0 ± 0.1	17.4 ± 0.4	21.5 ± 0.2	3.2 ± 0.5	24.7	21.1
1 mm, SHS 190 °C 60 min, 400 U/g laccase	46.9 ± 1.2	16.5 ± 0.6	19.4 ± 1.2	3.1 ± 0.0	22.5	28.1
0.5 mm, SHS 190 °C 60 min, 400 U/g laccase	48.5 ± 0.0	15.7 ± 1.1	16.9 ± 1.3	3.1 ± 0.0	20.0	36.1
0.25 mm, SHS 190 °C 60 min, 400 U/g laccase	49.3 ± 1.5	15.0 ± 1.2	15.8 ± 0.4	3.1 ± 0.0	18.9	39.6

**Table 3:** Lignin removal at different laccase loadings on oil palm empty fruit bunch (OPEFB) and oil palm mesocarp fiber (OPMF).

Samples	Components (%)			Lignin Removal (%)
	Insoluble Lignin	Soluble Lignin	Total Lignin	
Untreated OPEFB	22.6 ± 0.1	3.0 ± 0.0	25.6	-
20 U/g-substrate	21.5 ± 0.2	3.2 ± 0.0	24.7	3.5
40 U/g-substrate	21.0 ± 0.4	3.3 ± 0.4	24.3	5.0
60 U/g-substrate	20.4 ± 1.1	3.3 ± 0.1	23.7	7.4
80 U/g-substrate	20.1 ± 0.0	3.4 ± 0.0	23.5	8.2
100 U/g-substrate	19.6 ± 0.5	3.2 ± 0.2	22.8	10.9
200 U/g-substrate	19.6 ± 0.3	3.3 ± 0.0	22.9	10.5
Untreated OPMF	27.9 ± 0.1	3.4 ± 0.2	31.3	-
100 U/g-substrate	27.4 ± 0.4	3.3 ± 0.1	30.7	1.9
200 U/g-substrate	26.8 ± 1.1	3.1 ± 0.4	29.9	4.5
400 U/g-substrate	25.6 ± 0.1	3.1 ± 0.1	28.7	8.3
600 U/g-substrate	25.3 ± 0.1	3.3 ± 0.0	28.6	8.6
800 U/g-substrate	25.5 ± 0.3	3.2 ± 0.2	28.7	8.3

**Table 4:** Biological pretreatment using laccase on various lignocellulosic biomasses.

Substrates	Laccase Treatment	Laccase Loading (U/g-Substrate)	Lignin Removal (%)	References
Oil palm empty fruit bunch (OPEFB)	*P. sanguineus* UPM4	50	3.06	[22]
Wheat straw fiber	*P. cinnabarinus*	65	5.0	[27]
Furfural residue	*T. versicolor*	100	1.3	[28]
*Eucalyptus globules* kraft pulp	*T. vilosa*	17.5	23	[29]
Oil palm empty fruit bunch (OPEFB)	*T. versicolor*	100	10.9	This study
Oil palm mesocarp fiber (OPMF)	*T. versicolor*	400	8.3	This study
